# Prospective assessment of Y-chromosome microdeletions and reproductive outcomes among infertile couples of Japanese and African origin

**DOI:** 10.1186/1743-1050-2-9

**Published:** 2005-06-29

**Authors:** Paul E Kihaile, Atsushi Yasui, Yoshihiro Shuto

**Affiliations:** 1 Oita Medical University, Oita City, 879-5593 Japan; 2 St. Luke IVF Center, Oita City, 870-0497 Japan; 3 Muhimbili University College of Health Science, Box 65001, Dar Es Salaam, Tanzania

## Abstract

**Background:**

To compare the frequency of Y-chromosome microdeletions in Japanese and African azoospermic and oligozoospermic men and describe embryo characteristics and reproductive outcome following in vitro fertilization (IVF) with intracytoplasmic sperm injection (ICSI).

**Methods:**

Our study was performed prospectively at two centers, a private IVF clinic and a university hospital. Japanese and African (Tanzanian) men with nonobstructive azoospermia (NOA) and oligozoospermia (concentration < 5 × 10^6 ^/ml) were evaluated for Y-chromosome microdeletions (*n *= 162). Of the 47 men with NOA, 26 were Japanese and 21 were Africans. Of the 115 men with oligozoospermia, 87 were Japanese and 28 were Africans. Reproductive outcomes of patients with Y-chromosome microdeletions were then compared with those of 19 IVF+ICSI cycles performed on couples with Y-chromosome intact males/tubal factor infertility which served as a control group.

**Results:**

Seven azoospermic and oligozoospermic patients had Y-chromosome deletions; the total number of deletions in the AZFc region was five. There was only one deletion in the AZFa region and one complete deletion involving all three regions (AZFa, b, and c) within AZF. In our study population, microdeletion frequency among Japanese men was 6.2% (95% CI, 4.25% – 14.45%), whereas no deletions were identified in the African group (95% CI, 0.0% – 7.27%). The difference between the two groups was not statistically significant, however. Embryos derived from ICSI utilizing sperm with Y-chromosome microdeletion showed reduced rates of fertilization, blastocyst development, implantation, and pregnancy compared to the Y-chromosome intact group, although these observed differences were not statistically significant.

**Conclusion:**

The observed frequency of Y-chromosome microdeletion was 6.2% among Japanese azoospermic and oligozoospermic males; no microdeletions were identified among our African study patients. In this population of couples undergoing IVF+ICSI, there was no statistically significant difference in embryo characteristics or pregnancy outcome between patients with Y-chromosome microdeletion and those with an intact Y-chromosome.

## Introduction

Approximately15% of the couples worldwide cannot conceive after one year of regular sexual intercourse, with the male factor accounting for ~40% of all infertility; however, in up to 30% of cases the etiology is unexplained [1,2]. Among these unknown cases of infertility, a genetic etiology for male infertility was long suspected. This was due to cytogenic evidence showing that 0.2% of azoospermic men, who were otherwise phenotypically normal, exhibited Y-chromosome microdeletions [3]. This evidence was supported by karyotyping which revealed autosomal translocations in 1.3% of infertile couples [4-7]. Researchers realized that many cases of male infertility might be genetic because of the failure of most clinical treatments to correct abnormal sperm parameters [8,9]. Observations of sperm counts in various species, including studies of naturally occurring deletions in drosophila via molecular analysis, also led to an intense search for human spermatogenesis gene(s) which might be deficient in some infertile males [10-13]. Recent advancements in molecular methodology have permitted careful mapping of Y-chromosome microdeletions in men with azoospermia and oligozoospermia; in Western populations this frequency varies between 1–35%, depending on inclusion criteria [14]. For Japanese males, a Y-chromosome microdeletion frequency range of 7.6–17% has been reported [15-22]. Such studies have identified three "azoospermic factor" regions (AZF) where deletions occur on the Y-chromosome long arm: AZFa, AZFb, and AZFc. AZFc was shown to contain the most frequently deleted gene cluster, known as the DAZ gene [23]. Several studies found AZFc deletions to be associated with successful retrieval of sperm during testicular sperm extraction (TESE), whereas deletions in AZFa and AZFb were not [23-25]. Histologically, these deletions are associated with various spermatogenetic alterations including Sertoli cell-only syndrome (SCOS), maturation arrest, and hypospermatogenesis.

Recently, several investigators have shown that embryo characteristics following intracytoplasmic sperm injection (ICSI) using sperm obtained from men with Y-chromosome microdeletions were not adversely affected by the deletion [26-31]. The central concern was that vertical transmission of the microdeletion via ICSI might be passed from father to son [32] or by natural (unassisted) conception [30,33]. Since very few studies concerning Y-chromosome microdeletion have been undertaken in Japanese males (and none in African males), we aimed to investigate the frequency of Y-chromosome microdeletion as well as selected embryo features and reproductive outcome in Japanese and African azoospermic and oligozoospermic men who underwent IVF+ICSI.

## Materials and Methods

Between January 1998 and January 2003, male volunteers (*n *= 162) presenting for infertility evaluation and treatment at two centers were evaluated for Y-chromosome microdeletions via peripheral venipuncture. The study population consisted exclusively of Japanese (*n *= 113) and African (*n *= 49) males who, with their partners, sought infertility treatment either at St. Luke IVF Center (Japan) or Muhimbili National Hospital (Tanzania). Written informed consent was obtained from all study patients and the investigation was approved by the hospital's ethical committee. Subjects were partitioned into two groups based on sperm concentration: 1) nonobstructive azoospermia (NOA), and 2) oligozoospermia, defined as sperm concentration < 5 × 10^6 ^ml.

The first group consisted of 47 males (26 Japanese and 21 Africans); the second group consisted of 115 males (87 Japanese and 28 Africans). None of the study patients were diagnosed with obstructive azoospermia. Six couples from the Muhimbili center were excluded from the study due to active STD. The GFX Genomic Blood DNA Purification Kit (Amersham Biosciences, Buckinghamshire, United Kingdom) was used to extract DNA from peripheral venipuncture samples as previously described [34]. Y-chromosome microdeletions were detected using a polymerase chain reaction (PCR) amplification with a specific sequence tag site (STS) using 24 sets of primers which allowed evaluation of the following sites: sY14, sY18, sY78, sY81, sY83, sY85, sY84, sY90, sY100, sY131, sY134, sY139, sY145, sY143, sY153, sY147, sY156, sY149, sY254, sY157, sY202, sY243, sY158, and sY159. Deletion of the loci was confirmed if the product of the expected size was not obtained after three single STS PCR experiments.

Four patients with NOA and Y-chromosome microdeletion underwent TESE. The samples were microscopically examined to search for sperm, which was cryopreserved as described previously [24]. TESE was successful in 2 of 4 azoospermic cases; after cryopreservation these couples subsequently underwent two ICSI cycles. The three Japanese oligozoospermic patients with Y-chromosome microdeletion produced fresh ejaculated sperm which was subsequently used for 6 ICSI cycles. Sperm morphology in each laboratory was examined by 2 observers [35]; classification of normal sperm morphology at our centers is: <4% = severe teratozoospermia, 4–14% = moderate teratozoospermia and >14% = normal. No fathers or brothers of our male patients were available for testing. All Japanese couples who had a multifactoral diagnosis of oligozoospermia and tubal factor infertility were also assessed for Y-chromosome microdeletions and those found to have an intact Y-chromosome were assigned to the control group. Fourteen Japanese couples were initially considered for this group, but three were excluded because of endometriosis (*n *= 2) and leiomyoma (*n *= 1). These remaining couples (*n *= 11) constituted the control group and they underwent 19 ICSI cycles. Positive and negative controls were used for all AZF microdeletion tests. Thirteen fertile men with a sperm concentration of >20 × 10^6 ^/ml were used as positive controls, and twelve females served as negative controls.

Ovarian stimulation protocol, oocyte handling, laboratory procedures for insemination, measurement of sperm parameters, hormones, ICSI, and embryo and blastocyst grading were performed as previously described [35]. Using this protocol, only types I, II and III embryos were considered suitable for transfer and ≤ 3 embryos were transferred on day 3 after microinjection. For 6 cycles, embryos were cultured until day 5–6 and were transferred at the blastocyst stage. Clinical pregnancy was confirmed at 6 weeks via transvaginal ultrasound to establish embryonic cardiac activity.

Data were analyzed for equality of variance using the Levene's test. When p > 0.05 the variances were considered equal, a Student's t-test was performed, and p < 0.05 was considered significant. When p < 0.05, then the variances were considered unequal and a Wilcoxon signed ranked test was done, and p < 0.05 was considered significant. All computations were conducted using the SPSS statistical package (SPSS Inc., Chicago, USA).

## Results

Of 162 azoospermic and oligozoospermic Japanese and African males who participated in the study, 47 were diagnosed with NOA and 115 as oligozoospermic. Of 47 NOA cases, 26 were Japanese and 21 were Africans. Of 115 oligozoospermic cases, 87 were Japanese and 28 were African. We identified seven cases with microdeletions and they were all within the azoospermia and oligozoospermia Japanese group. Five of these deletions were identified in the AZFc region, whereas only one deletion was identified in the AZFa region. There was also one complete deletion involving all three regions of AZF (AZFa, b, c). Therefore, the microdeletion frequency within the Japanese group was 6.2% (95% CI, 4.25% – 14.45%). No deletions were identified within the African group (95% CI, 0.0% – 7.27%); this difference was not statistically significant. The deletion frequency among azoospermic Japanese males was 15.4% (95% CI, 11.0% – 42.0%) whereas there were none among the African males (95%CI, 0.0% – 15.4%). Again, the difference did not reach statistical significance. The deletion frequency in oligozoospermic Japanese was 3.4% (95% CI, 1.18% – 9.6%) and there were none in oligozoospermic African males (95% CI, 0.0% – 12.0%); also with no statistical difference.

Two patients were diagnosed with SCOS and both exhibited one microdeletion in the AZFa and AZFabc regions. (Table 2). These two patients had characteristically high serum FSH levels (mean = 30.4 U/L, normal is < 10.0 U/L in our laboratory). Testicular volumes of males with Y-chromosome microdeletion and those with an intact Y-chromosome showed no statistically significant difference (average ± SD volumes were 11.14 ± 2.41 and 12.4 ± 3.40 ml, respectively).

Table 3 summarizes comparisons among various parameters in patients with and without Y-chromosome microdeletion. As expected, sperm concentration was significantly lower in the Y-chromosome microdeletion group than in the intact Y-chromosome group (*p *< 0.05).

Table 4 presents a comparison of embryo characteristics observed in AZFc microdeletion and Y-chromosome intact patients. Although the Y-chromosome microdeletion group showed a trend towards reduced rates of fertilization, implantation, and pregnancy when compared to the Y-chromosome intact group, this difference did not reach statistical significance. Screening for Y-chromosome microdeletions among our study patients' male offspring was not conducted in this investigation.

## Discussion

This study shows the frequency of microdeletion in the AZF region of the Y-chromosome to be 6.2% among Japanese males with azoospermia and oligozoospermia. Our findings are consistent with prior reports which found a microdeletion frequency of 1% to 35%, depending on the male subfertility definition used for inclusion and on the choice of sequence tagged sites used for screening [6,14,28,36,37]. In our populaton, the majority of Y-chromosome microdeletions (~70%) occurred in the AZFc region of the AZF region and is in agreement with other investigations [27,38]. Previous studies on Y-chromosome microdeletion frequency in Japanese males [15-22] suggested a range of 7.6% – 17.0%. Separating these Japanese patients with deletions into azoospermic and oligozoospermic cases revealed frequencies of 15.4 % and 3.4%, respectively.

We excluded from analysis two study patients with Y-chromosome microdeletions at sY202 and sY243. Indeed, no known location has been ascertained so far for sY202 and sY243 is found in several locations both inside and outside DAZ. Nevertheless, the frequency observed in our population is consistent with previous data describing a 15%-20% microdeletion frequency in men with idiopathic azoospermia and a 7%-10% frequency in men with severe idiopathic oligozoospermia [15,23,38,39]. Interestingly, no microdeletions were documented among the 49 African males with azoospermia or severe oligozoospermia. Since ours is the first study of Y-chromosome microdeletion frequency to be undertaken on an African population, a comparison with earlier data sets from this group was not possible.

The absence of Y-chromosome microdeletions in our African study population may be due to limited sampling. However, this finding is in general agreement with the few studies conducted in other parts of Africa that found frequencies of male infertility secondary to oligozoospermia or azoospermia to be much lower (~20%) in Africa than elsewhere [40-48]. Unlike some other regions, the most common cause of male infertility in Africa was found to be infection. Yeboah *et al*. reported on 595 infertile African males and found ~70% of them to have inflammatory testicular lesion due to STD [40]. A multi-center study by Cates *et al*. demonstrated that >50% of African couples had secondary infertility due to STD [41], which was a rate much higher than in non-African countries (*i.e*., <30%).

In our study all active STD cases were excluded, although the association between male infertility and STD remains controversial. Some investigators have shown that treatment of infection directly improved the sperm quality in oligozoospermia [42,43] while others did not see any improvement in sperm quality after treatment [44,45]. It is difficult to know the precise frequency of azoospermia and oligozoospermia in Africa as certain cultural factors (*e.g*., polygamy) are more common than in other parts of the world [46]. Therefore, an azoospermic man may have children whose actual biological father was another man if the wife had extramarital intersourse with a fertile male [47,48]. Financial constraints did not enable testing to confirm paternity when babies were born after infertility treatments at our centers.

All 5 cases with Y-chromosome microdeletion in the AZFc region had successful outcomes following IVF+ ICSI. A comparison of reproductive outcomes between couples with AZFc microdeletion and couples with an intact Y-chromosome showed no statistically significant difference. Our results confirm previous studies showing that Y-chromosome microdeletions do not appear to adversely affect fertilization and pregnancy rates (either in azoospermic or severe oligozoospermic men) when sperm are successfully retrieved [26-33,36]. The concerns that IVF+ICSI might yield poorer results in the setting of Y-chromosome microdeletions were not seen in previous reports [26-30]. However, Van Volde *et al*. [29] found fertilization and embryo quality to be significantly lower in couples with Y-chromosome microdeletions compared to couples without Y-chromosome microdeletions; pregnancy and take home baby rates were not statistically different.

In conclusion, our investigation did not detect any Y-chromosome microdeletions in azoospermic or severely oligozoospermic men of African origin. This was in contrast to seven cases of Y-chromosome microdeletion identified in Japanese males. Furthermore, comparison of embryo characteristics in Japanese couples with Y-chromosome microdeletion and control couples (those with no Y-chromosome microdeletion) revealed no statistically significant difference. We regard it as premature to conclude definitively that Y-chromosome microdeletion in azoospermic and oligozoospermic African males is not as common as in other races, due to limited sampling. We hope to continue this investigations with a larger patient population to provide additional information on the overall frequency of azoospermia/oligozoospermia in African males, as well as the association of these conditions with Y-chromosome microdeletions.

**Table 1:** STS markers of the 7 infertile men with Y chromosome deletions.

STS	Region							
Markers		1.1	1.2	1.5	1.6	1.7	1.8	1.9

sY14		+	+	+	+	+	+	+
sY18		+	+	+	+	+	+	+
sY78		+	+	+	+	+	+	+
sY81	AZFa	-	-	+	+	+	+	+
sY83		-	-	+	+	+	+	+
sY85		-	-	+	+	+	+	+
sY84		-	-	+	+	+	+	+
sY90		-	-	+	+	+	+	+
sY100	AZFb	+	-	+	+	+	+	+
sY131		+	-	+	+	+	+	+
sY134		+	-	+	+	+	+	+
sY139		+	-	+	+	+	+	+
sY145		+	-	+	+	+	+	+
sY143		+	+	+	+	+	+	
sY153	AZFc	+	-	-	-	-	-	-
sY147		+	-	-	-	-	-	-
sY156		+	-	-	-	-	-	-
sY149		+	-	-	-	-	-	-
sY254		+	-	-	-	-	-	-
sY157		+	-	-	-	-	-	-
sY202		+	-	-	-	-	-	-
sY243		+	-	-	-	-	-	-
sY158		+	-	+	+	+	+	+
sY159		+	-	+	+	+	+	+

**Table 2:** Findings in 7 infertile men with Y chromosome microdeletion

	Patient Nos.						
	
	1.1	1.2	1.5	1.6	1.7	1.8	1.9
Left/right testis volume (ml)	9&14	12&14	14&15	13&15	9&8	13&14	8&7
sperm conc (× 10^6 ^/ml)	0	0	0	0.9	0.6	1.5	0
Deleted AZF regions	a	a, b, c	c	c	c	c	c
FSH (U/ml)	29.5	31.3	10.3	11.6	9.1	12.7	14.3
Testicular histology	SCOS	SCOS	MA	ND	ND	ND	MA
Sperm testicular recovery	0	0	+	ND	ND	ND	+

ND = Not done; MA = Maturation Arrest; SCOS = Sertoli cell only syndrome

**Table 3:** Results of the various parameters of ejaculate sperm cycles in patients with AZFc region Y-deleted and Y-Intact chromosomes.

	AZFc region		
	Y-deleted Chromosome	Y-Intact Chromosome	Significance
Total No. Patients	5	11	
Total No. Cycles	8	19	
Female Age (mean)	30.9 ± 6.2	30.4 ± 5.7	NS
Duration of infertility in years (mean)	4.9 ± 3.1(2–10)	4.4 ± 2.7(2–6)	
Peak E2 pg/dl (mean)	3613 ± 1876	3785 ± 1543	NS
Embryos Transferred (average))	2.7(2–3)	2.8 (2–4)	NS
Sperm Normal Morphology (%)	2.3 ± 0.5%	3.4 ± 0.7	NS
Sperm Concentration (× 10^6^/mL)	1.5 ± 1.1	4.3 ± 1.2	*p *< 0.05
Motility (%)	35 ± 14.7	41 ± 11.4	NS

**Table 4:** ICSI attempts in 8 cycles with Y-chromosome microdeletions and 19 cycles without microdeletions.

	Y-deleted	Y-Intact	
	Chromosome	Chromosome	Significance

No cycles with ejaculated sperm	6	19	NS
No. cycles with Testicular sperm	2	0	
Total No. oocytes	108	269	NS
Average No.oocytes (n)	13.5 ± 2.7	12.8 ± 3.5	NS
Fertilization rate(%)	60.1 ± 17.9%	71.6 ± 15.7%	NS
Cleavage rate (%)	87.6 ± 14.5	85.1 ± 9.4	NS
Embryo grade 1 & II on day 3/cleaved embryos	51.70%	59.60%	NS
≥6 cells embryos on day 3/cleaved embryos (%)	75.00%	72.9	NS
Blastocyts on day 5&6 /cleaved embryos (%)	50.00%	53.40%	NS
Canceled cycle(s)	1	0	NS
Average mixed embryo & blastocyst ET	2.7 ± 0.5	2.8 ± 0.9	NS
Pregnancy	3 (37.5%)	9 (42.9)	NS
Implantation rate	13.5	16.9	NS
